# Impact of plastic mulch on soil microbial abundance, activity, and diversity in the saline-alkali soils of Xinjiang

**DOI:** 10.3389/fmicb.2025.1691334

**Published:** 2025-11-26

**Authors:** Changxue Wu, Junxiao Zhang, Yongmei Xu, Xiangwen Xie

**Affiliations:** Institute of Agricultural Resources and Environment, Xinjiang Academy of Agricultural Sciences, Urumqi, Xinjiang, China

**Keywords:** saline-alkali land, biodegradable plastic film, polyethylene film, soil microorganisms, cotton fields

## Abstract

**Introduction:**

As biodegradable plastic (BP) film gradually replaces traditional polyethylene (PE) film as an environmentally friendly alternative in agriculture, its impact on the soil microbial environment has attracted considerable attention.

**Methods:**

This study examined the effects of different film coverings on the quantity, activity, and diversity of soil microorganisms by comparing fully biodegradable films with thicknesses of 0.01 mm and 0.012 mm to conventional PE films in typical cotton fields of Xinjiang.

**Results:**

Results showed that the overall trends in microbial population dynamics were generally consistent across treatments. The abundance of bacteria and actinomycetes was highest under PE film, while fungi abundance peaked under the 0.012 mm BP film. With longer cultivation time, soil microorganisms exhibited a significant increase in total carbon source metabolism. Across the entire cotton growth period, different mulching treatments had only minor effects on microbial diversity. Both film cracking rates and the local environmental conditions emerged as key factors influencing the soil microbial community.

**Conclusions:**

Considering the need for a low cracking rate during the seedling stage as well as soil environmental sustainability, BP film with a thickness of 0.01mm appeared to be the most suitable degradable option for practical use in cotton fields. Nevertheless, comprehensive evaluation of its long-term environmental benefits will require further research and sustained monitoring in conjunction with cotton growth.

## 1 Introduction

Xinjiang is not only a major grain production base in western China but one of the world’s key grain supply regions (Lu et al., 2018). However, the region faces severe ecological and environmental challenges, particularly frequent extreme droughts and severe soil salinization, which have resulted in substantial declines in crop yield and quality (Long et al., 2021). Statistics indicated that millions of tons of crop yield were lost annually in Xinjiang due to these stresses, posing a serious threat to the sustainable development of local agriculture. Against this backdrop, identifying effective agricultural technologies to improve the productivity of saline-alkali land is of critical importance. As a traditional agronomic practice, crop mulching has gained widespread attention in both domestic and international agricultural research and production (Gao et al., 2019). By covering the soil surface with film materials, mulching can effectively reduce water evaporation, conserve soil moisture and nutrients, and suppress the spread of pests and diseases, thereby improving crop yield and stress resistance (Nawaz et al., 2017).

Over the past three decades, the extensive use of plastic film has made it a pillar technology in regional agriculture (Gu et al., 2020), with an annual consumption of approximately 1.4 million tons and a coverage area of 18.4 million hectares (Gao et al., 2019; Li et al., 2016). However, the widespread application of high-strength films has also given rise to a series of ecological problems, including residual film pollution, soil compaction, and fertility decline (Koskei et al., 2021). Different types of plastic films are suit to varying agricultural practices and environmental needs, with each type characterized by distinct raw materials and degradation mechanisms. Conventional plastic films are difficult to degrade under natural conditions, and their long-term accumulation has led to severe soil residue problems. These residues adversely affect soil structure, porosity, and permeability, thereby inhibiting microbial growth and activity and ultimately disturbing soil ecological balance (Hao et al., 2024; Ren et al., 2021).

As an alternative, degradable films have gained increasing attention due to their environmentally friendly and biodegradable properties (Zhang et al., 2024). Among them, biodegradable plastic (BP) films have emerged as a promising substitute for polyethylene (PE) films. They are easy to apply, deliver comparable agronomic benefits, degrade *in situ*, and contribute to improving soil fertility (Akhir and Mustapha, 2022). In agricultural production, BP films gradually break down under the combined influence of soil microorganisms and natural factors such as light and heat. And they can be directly degraded in the soil without requiring recycling (Shen et al., 2019; Xochitl et al., 2021), effectively mitigating the soil compaction and fertility decline associated with traditional plastic films (Hao et al., 2024; Ren et al., 2021). Importantly, BP films do not generate residual pollution, making them particularly suitable for arid regions and supportive of sustainable agroecosystem development (Deng et al., 2019). With the advancement of agricultural modernization and the growing emphasis on sustainability in the context of urbanization, the demand for BP films is expected to continue increasing (Zhang L. et al., 2020).

Due to differences in degradation mechanisms and material properties, various types of degradable plastic films exert distinct impacts on the soil microbial environment (Bai et al., 2024; Bandopadhyay et al., 2020). Compared with non-biodegradable mulch films, BP films have been reported to generate higher environmental impacts across most life cycle assessment categories (de Sadeleer and Woodhouse, 2023). Nevertheless, BP mulching can effectively increase soil temperature at depths of 5, 15, and 25 cm throughout the entire maize growth period (Zhang W. W. et al., 2020), and has shown comparable performance to PE films in promoting crop growth, yield, and water use efficiency under arid conditions (Deng et al., 2019). Zhao et al. (2022) further demonstrated that BP films are slightly inferior to traditional white mulch films in terms of mechanical strength, hydrophobicity, insulation, and moisture retention. However, they outperform conventional films in terms of aging resistance, soil oxygen content, and crop insulation and water storage capacity during the middle and late growth stages. In addition, Chen L. Y. et al. (2023) reported that the abundance of microplastics in farmland soils varies with crop type, being highest in fruit fields and lowest in economic crop fields, with cotton and maize fields showing the lowest levels. These findings highlight that despite the potential benefits of BP films, their widespread adoption still faces challenges, and a comprehensive and rigorous evaluation of their environmental impacts remains essential (Yang et al., 2020). Importantly, once degraded, BP films not only avoid causing ecological harm but can also contribute to the biological metabolic cycle and enhance soil microbial activity (Guan et al., 2022).

Soil microorganisms are a vital component of soil ecosystems, playing an essential role in material transformation, nutrient cycling, and energy flow (Gavazov et al., 2017). The effects of plastic film mulching on the soil microbial environment are multifaceted. On the one hand, mulching can increase soil temperature and moisture, thereby improving microbial habitats and stimulating microbial growth and metabolism. On the other hand, it alters microbial community composition and spatial distribution by influencing soil pH, aeration, carbon dioxide concentration, and enzyme activity (Wang et al., 2021). Previous studies have reported that mulching treatments can increase the number of bacterial OTUs, while other findings suggest that degradation intermediates may temporarily inhibit the activity of certain microbial groups (Liu et al., 2012). Although crop mulching has been widely recognized for its potential to improve agricultural productivity in arid environments, its application in saline-alkali soils remains insufficiently understood. Saline-alkali soils are characterized by high salt content, alkaline pH, and low fertility, which may influence the adhesion and water retention performance of mulching films. Moreover, there is still a lack of systematic research on how mulching affects on soil microbial communities in saline-alkali soils, particularly regarding the regulation of microbial structure and function.

In this context, the present study investigated the effects of mulching films on soil microbial quantity, metabolic activity, and diversity under saline-alkali conditions. The findings are expected to provide a scientific basis for improving saline-alkali land management and advancing agricultural technology in Xinjiang, thereby enhancing crop yield and quality while contributing to regional food security.

## 2 Materials and methods

### 2.1 Experimental materials and design

The field experiments were conducted in Changji City and Hutubi County, Xinjiang, China. The basic information on cotton planting areas at the two sites was provided in Table 1. The BP films used in the experiment were fully biodegradable transparent polybutylene adipate terephthalate (PBAT) mulch films, produced by Xinjiang Lanshan Tunhe Chemical Co., Ltd. Two BP film treatments with thicknesses of 0.01 mm (T1) and 0.012 mm (T2) were established, each with three replicates. The control (CK) consisted of conventional polyethylene (PE) film with a thickness of 0.008 mm, which was the most widely used material in local agricultural production (Xu et al., 2023).

**Table 1:** Cotton planting area at different experimental locations.

Site	Biodegradable mulch film		Ordinary PE film (CK)
	0.01 mm (T1)	0.012 mm (T2)	
Changji	1.67 ha	1.67 ha	3.34 ha
Hutubi	1.67 ha	1.67 ha	3.34 ha

### 2.2 Sample collection and processing

Soil samples were collected for each treatment according to the growth period of crops, and the sampling time is shown in Table 2, representing the seedling stage, bud stage, boll stage, and boll opening stage of cotton, respectively. Each soil sampling point should meet the conditions of flat terrain and consistent crop growth. And it should form an isosceles or equilateral triangle with two adjacent plants on the ground. Then collect soil samples 30 cm below the film, mix 3–4 holes of soil evenly, and discard stones, plant roots and other debris. Take three samples for each treatment. Then pack and seal the soil samples with labels, and place in a refrigerator at 4 °C after returning to the laboratory.

**Table 2:** Sampling schedule.

Sample time	1	2	3	4	5
Changji	5/27	8/19	9/10	9/29	\
Hutubi	5/26	7/13	8/10	9/10	9/29

### 2.3 Data analysis

#### 2.3.1 Count of major microbial groups

Using the plate counting method with nutrient agar, Gao Shi No.1, and Bengal Red medium, bacteria, actinomycetes, and fungi were effectively isolated and accurately quantified through gradient dilution, plating, and constant-temperature incubation. Colony counts were considered valid when bacterial and actinomycete plates contained 20–200 colonies and fungal plates contained 10–100 colonies. The average colony number per plate was then calculated for each group.

#### 2.3.2 Carbon source metabolism and utilization of microbial communities

Using the Average Well Color Development (AWCD) value of soil microbial communities to reflect information on microbial community activity, carbon source utilization efficiency, community differences, and ecological functions (Gao et al., 2020). The testing steps are as follows: weigh 1.0 g of fresh soil sample and add it to 10 mL of sterilized physiological saline (0.85%), shake well, let it stand for 20 min, and then dilute the soil sample to 1,000 times. Use an eight channel pipette to aspirate the diluted bacterial solution and add it to Biolog Eco microplates, adding 150 μL per well. Then place the microplate in a 30 °C constant temperature incubator for incubation. Use Biolog analysis and identification system for sample determination, read the plate once every 24 h for 7 consecutive days. The calculation formula for AWCD value is as follows (Gao et al., 2020):

$$\text{AWCD}=\sum (C_{i}-R_{i})/n$$

Where *n* represent the number of pores in the culture medium, and the *n* value in the Biolog ECO plate was 31 (that is 31 carbon sources, each with three repeating pores, usually calculated as an average); *C_i_* is the optical density value of i-th pore; *R_i_* is the optical density value of the control pore in the Biolog ECO plate. For pores with the difference between C*i* and *R_i_* less than zero, they are recorded as zero in the calculation, i.e., *C_i_*-*R* ≥ 0. In Biolog ECO plates, each well represents a type of carbon source, so AWCD values can also indirectly reflect the utilization efficiency of microbial communities toward various carbon sources. The larger the AWCD value, the stronger the microbial community’s ability to utilize carbon sources, indicating higher microbial activity.

#### 2.3.3 Microbial diversity analysis

Using Shannon Wiener diversity index (H), Simpson dominance index (D), and Mclntosh richness index (U) to represent the carbon source metabolic diversity of soil microbial communities, and calculate each index according to the method of Classen et al. (2003):

$$H=\sum P_{i}\times \ln(P_{i})$$

$$D=1-\sum P_{i}^{2}$$

$$U=\sqrt{\left(\sum n_{i}^{2}\right)}$$

where *P_i_* is the ratio of the relative absorbance value of the *i*-th well to the total relative absorbance value of the entire plate; *n_i_* is the relative absorbance value of the *i*-th hole.

## 3 Results

### 3.1 Changes in the quantity of soil microorganisms

**Figure 1:**
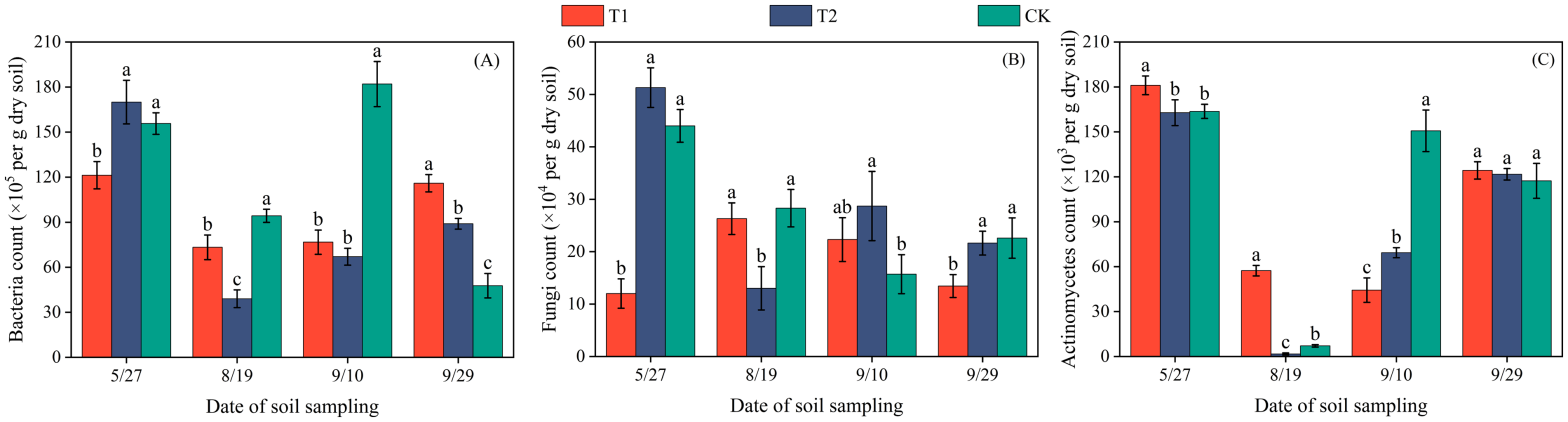
Bacterial **(A)**, fungal **(B)**, and actinomycete **(C)** counts per gram of dry soil in cotton fields of Changji. Error bars represent standard deviation. Different lowercase letters indicate significant differences among treatments at the 0.05 level (*p* < 0.05); identical letters indicate no significant difference.

**Figure 2:**
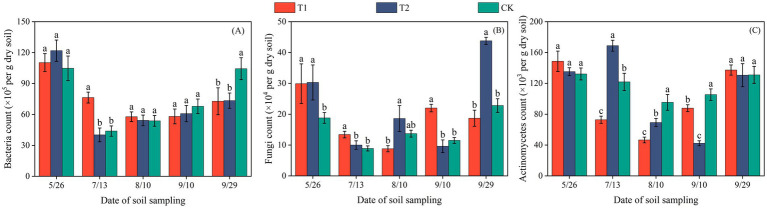
Bacterial **(A)**, fungal **(B)**, and actinomycete **(C)** counts per gram of dry soil in cotton fields of Hutubi.

The population size of soil microorganisms was presented as the number of colony-forming units per gram of dry soil (cfu·g^−1^). In both Changji and Hutubi cotton fields, bacterial abundance exhibited a trend of initially decreasing and then increasing during the early growth stages (Figures 1, 2). In Changji, before the third sampling (September 10), bacterial numbers first declined and then rose under all three treatments. After that, bacterial abundance continued to increase under T1 and T2 but declined to a lower level under CK. In Hutubi, bacterial abundance under CK and T2 began to increase after the second sampling (July 13), whereas under T1 it began to rise only after the third sampling (August 10). By the end of the reproductive stage, bacterial abundance under T1 and T2 remained at comparable levels, while CK maintained a higher abundance than both. Overall, CK had the highest bacterial abundance in Changji with an average value of 119.93 × 10^5^, whereas in Hutubi bacterial abundance was the lowest under T2, with an average value of 70.1 × 10^5^.

Fungal abundance displayed markedly different patterns across treatments. In Changji, under T1, fungi first increased and then decreased, while under CK the opposite trend was observed. The turning points of these changes occurred at the second sampling (August 19) and the third sampling (September 10), respectively. Under T2, fungal abundance followed a decrease–increase–decrease pattern. In Hutubi, fungal abundance fluctuated throughout the entire growth period, with CK and T2 showing generally consistent trends, though fluctuations were more pronounced under T2. After the fourth sampling (September 10), fungal abundance increased under T2 and CK but decreased under T1. Overall, T1 had the lowest fungal abundance in Changji, with an average value of 18.4 × 10^4^, whereas in Hutubi fungi reached their highest abundance under T2, with an average value of 22.46 × 10^4^.

In Changji, actinomycetes decreased initially and then increased under all treatments after the third sampling. Subsequently, their abundance declined under CK but continued to rise under T1 and T2. In Hutubi, actinomycetes showed comparable abundances during the early and late stages across all treatments. Under T1 and CK, they followed a decrease–increase pattern with a turning point at the third sampling (August 10). In contrast, under T2, actinomycetes displayed a sequence of increase–decrease–increase. Overall, actinomycete abundance was lowest under T2 in Changji, with an average value of 88.93 × 10^3^, while it was highest under CK in Hutubi (average: 98.64 × 10^3^).

Across both sites, the combined abundance of bacteria, fungi, and actinomycetes showed clear treatment-specific differences. In Changji, microbial community responses varied by group, with CK favoring bacterial abundance, T1 limiting fungi, and T2 suppressing actinomycetes. In Hutubi, the overall abundance followed the order T2 > T1 > CK, indicating a stronger stimulatory effect of T2 on total microbial activity.

### 3.2 Changes of AWCD

**Figure 3:**
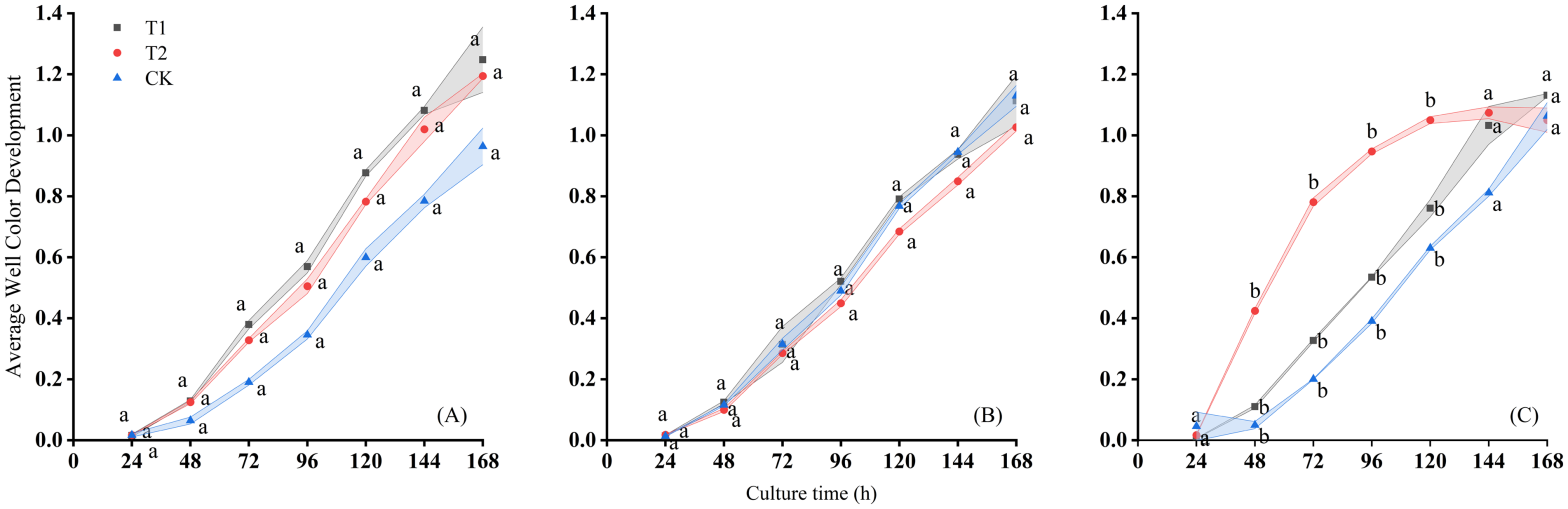
Changes of AWCD with culture time on the **(A)** August 19th, **(B)** September 10th, and **(C)** September 29th, 2016 in Changji cotton fields.

**Figure 4:**
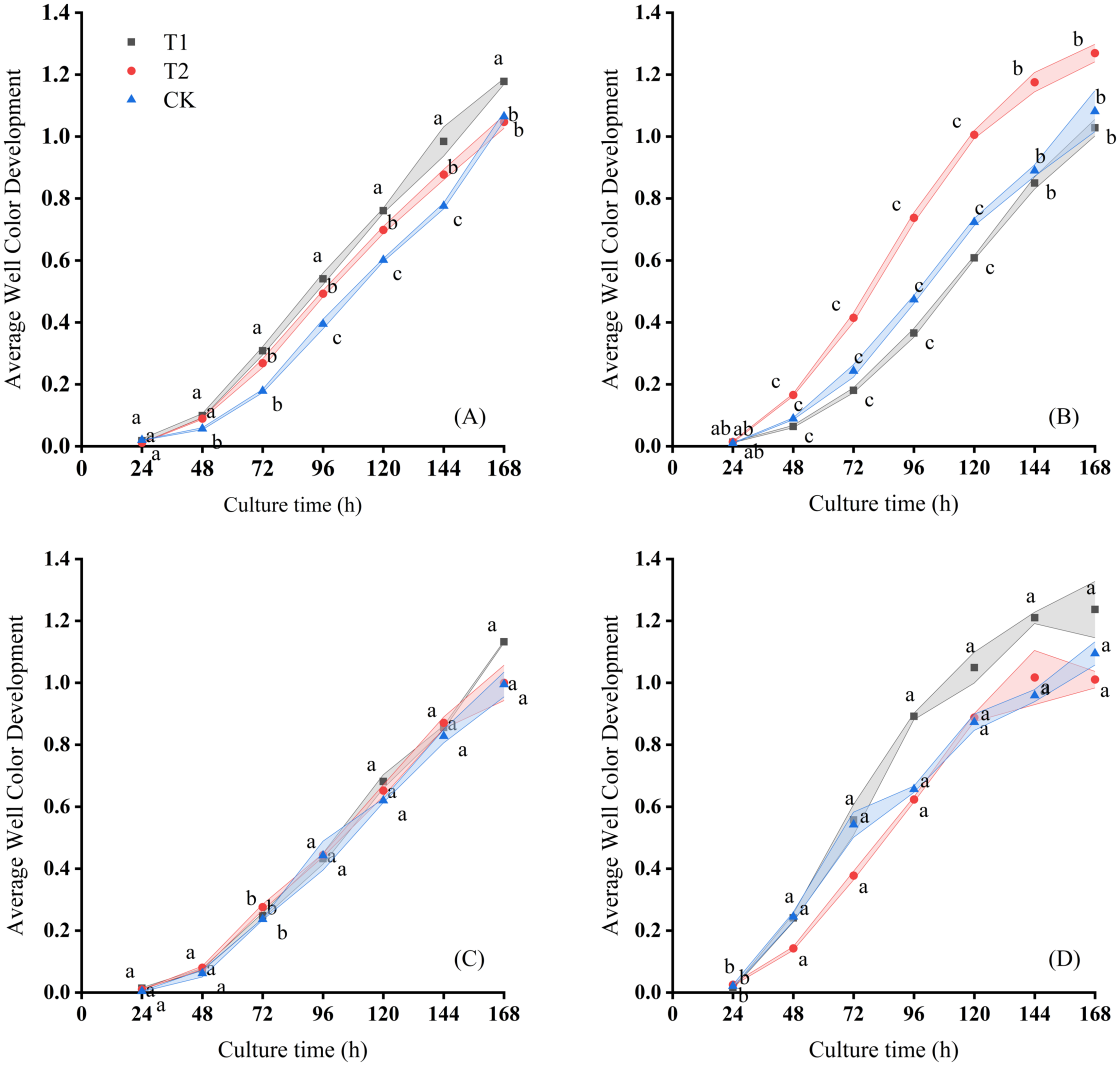
Changes of AWCD with culture time on the **(A)** July 13th, **(B)** August 20th, **(C)** September 10th, and **(D)** September 29th, 2016 in Hutubi cotton fields.

Under different treatments, the total metabolic activity of soil microorganisms toward various carbon sources increased markedly after 24 h of incubation in both sites (Figures 3, 4). In the Changji cotton fields, after 168 h of cultivation, the overall AWCD values of the treatments were ranked as T1 > T2 > CK on August 19, 2016; CK > T1 > T2 on September 10, 2016; and T1 > T2 > CK on September 20, 2016. The AWCD trends varied across sampling periods and treatments. On September 10 and September 20, AWCD values were lower compared with those on August 10. Across all periods, AWCD under the CK treatment was generally lower than under T1 and T2. The temporal dynamics of AWCD showed a consistent pattern of gradual increase with incubation time; however, on the September 20, the AWCD under T2 stabilized after 120 h of cultivation.

The microbial metabolic activity of samples from the Changji cotton field also increased significantly after 24 h of incubation. After 168 h, AWCD values in all treatments reached their maximum and then stabilized, with final values ranging between 1.3 and 1.4 (Figure 4). At 168 h, the ranking of AWCD values was: T1 > T2 > CK on July 13; T2 > CK > T1 on August 20, 2016; T1 > T2 > CK on the September 10, 2016; and T1 > CK > T2 on September 29, 2016. The AWCD trends differed across sampling periods and treatments, with the lowest values observed on September 10. As in Changji, AWCD under the CK treatment was generally lower. On the September 29, 2016, AWCD under T2 also tended to stabilize after 120 h of cultivation.

### 3.3 Changes of soil microbial diversity

**Figure 5:**
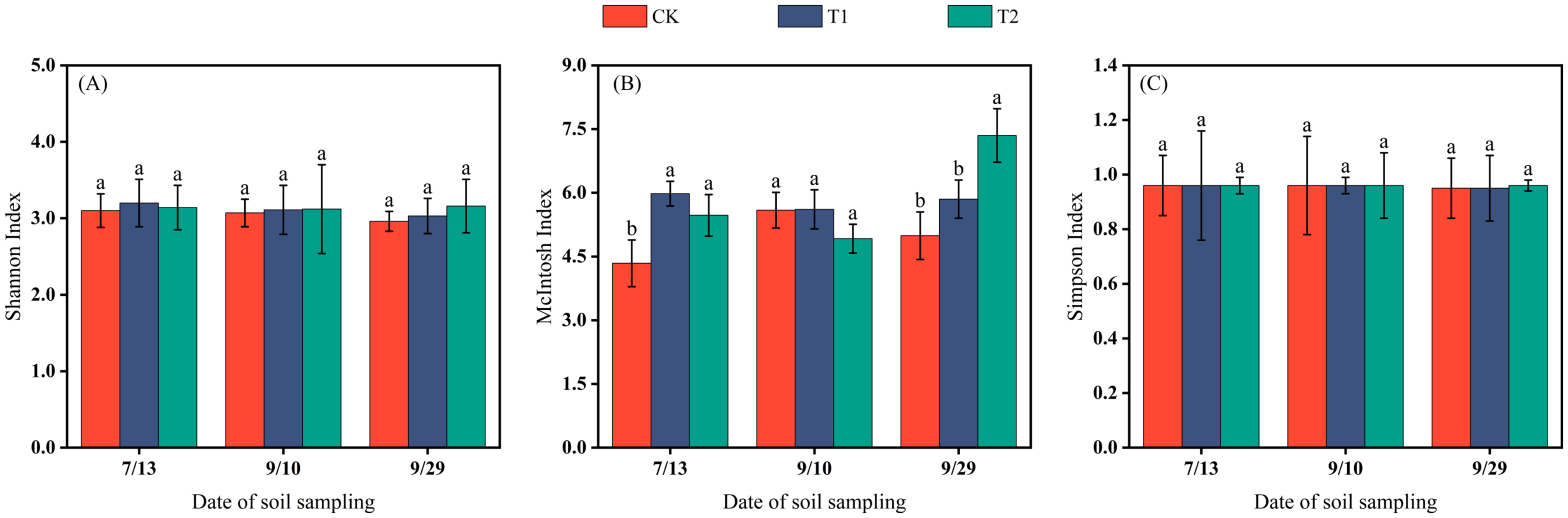
The Shannon index **(A)**, McIntosh index **(B)**, and Simpson index **(C)** of soil microorganisms in Changji cotton fields.

The differences in the Shannon and Simpson indices among treatments were not significant, indicating that mulching methods had no marked effect on the richness or dominance of soil microbial communities at different cotton growth stages. In contrast, the McIntosh index exhibited a wider range of variation (4.34–7.35). By the end of the reproductive stage, the McIntosh index under T2 was significantly higher than under the other two treatments (*p* < 0.05). Over the sampling period, the diversity indices of CK and T1 showed a decreasing trend, whereas under T2 they increased, suggesting that coverage with 0.012 mm biodegradable film promoted microbial diversity in the Changji fields during the late stage of cotton growth (Figure 5).

**Figure 6:**
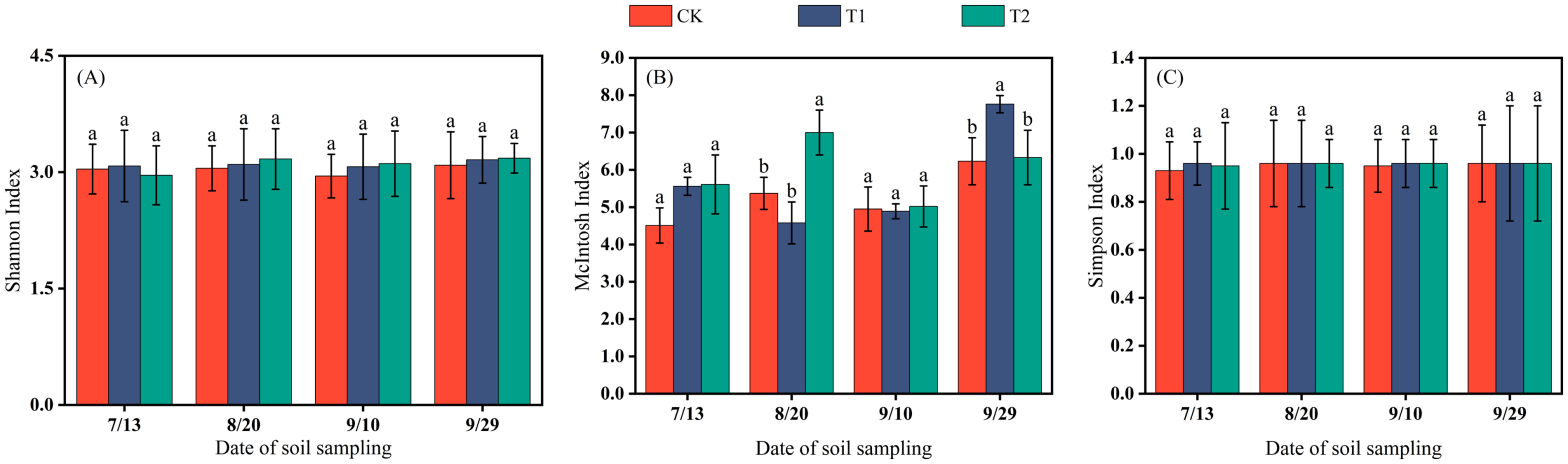
The Shannon index **(A)**, McIntosh index **(B)**, and Simpson index **(C)** of soil microorganisms in Hutubi cotton fields.

In the Hutubi cotton fields, changes in microbial diversity indices were generally consistent with those observed in Changji. The McIntosh index fluctuated more strongly than the Shannon and Simpson indices, and microbial diversity in the late growth stage was higher than in the early and middle stages (Figure 6).

## 4 Discussion

### 4.1 Impact of plastic film covering on soil microbial environment

In environmental impact and risk assessment, the degradation rate of BP films is considered more critical than their overall degradation capacity, as the effects of plastic particles on soil health, crop yield, and field characteristics remain unclear (Ferreira-Filipe et al., 2022). Previous studies have suggested that prolonged degradation periods may exacerbate negative impacts (Sintim et al., 2019).

**Figure 7:**
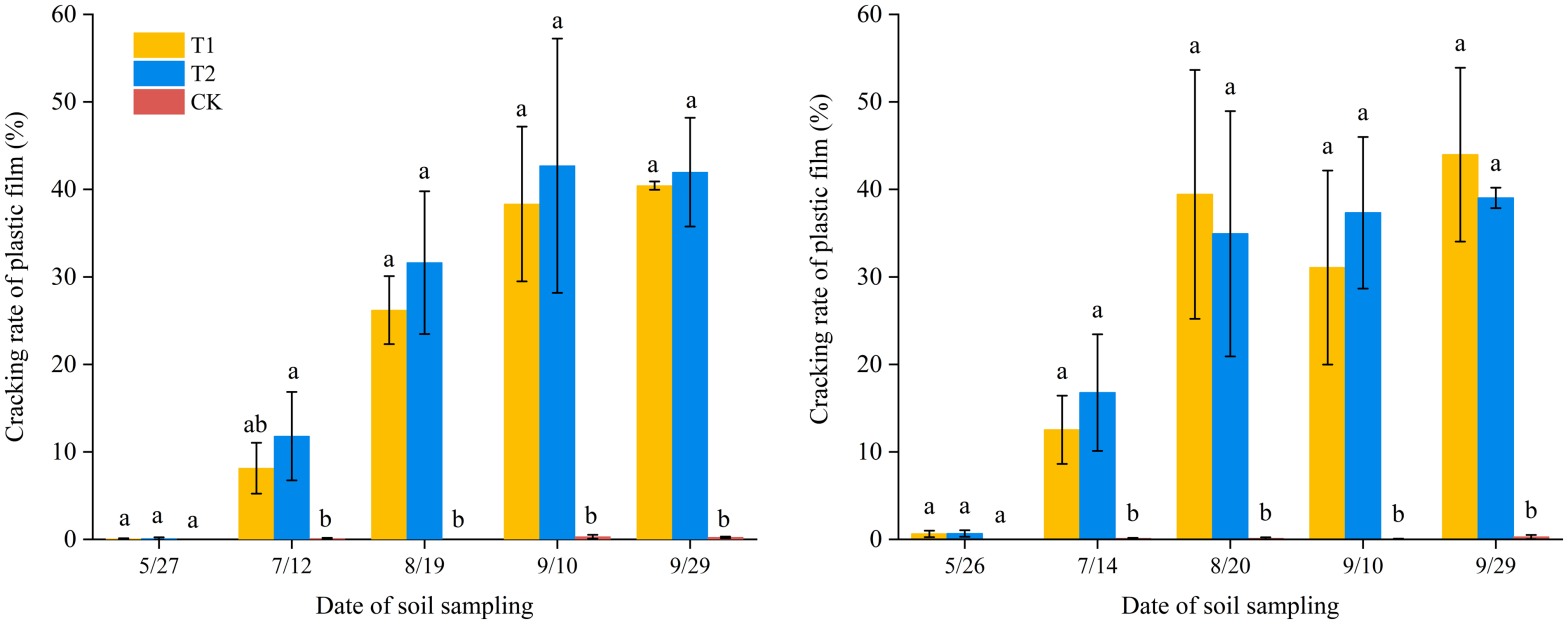
Cracking rate of plastic film in Changji and Hutubi cotton fields under different treatments.

In this study, the rapid extraction software V3.0 was applied to analyze the pore area of plastic films and quantity cracking during the cotton growth period (Figure 7). The results showed that, unlike BP films, conventiomal PE films exhibited no cracking throughout the entire growth period. Among the BP films, the 0.012 mm treatment displayed a significantly higher cracking rate than the 0.01 mm treatment. Overall, cracking rates increased with mulching duration. However, statistical analysis indicated no significant difference in cracking rates between BP films and PE films (*p* < 0.05). The maximum cracking rates of BP films were 40.4 and 42.7% in Changji, and 44.0 and 39.0% in Hutubi, for the two thicknesses, respectively.

Previous studies have reported that BP films may leave microplastics in the soil, which can accumulate with repeated application (Qi et al., 2021). Incomplete degradation of these residuals may affect crop growth, reduce yields, and impair soil quality (Li et al., 2020; Qi et al., 2020). In this study, the AWCD of soils covered with BP films was higher than that under PE films, indicating that BP films have the potential to enhance soil microbial activity. The 0.01 mm biodegradable film significantly promoted the growth of bacteria, fungi, and actinomycetes due to faster degradation, release of available carbon sources, and improved soil aeration and permeability; In contrast, the degradation of 0.012 mm film was slightly slower and the stimulating effect was weaker, while ordinary polyethylene film hardly degrades, so there was a significant difference in microbial abundance among the three. Moreover, the impact of mulching treatments on soil microbial quantity varied between regions. Significant differences in microbial populations were observed in the soils of Changji and Hutubi under different treatments (Figures 1, 2). In practical production, as plastic film crack, their degradation products may serve as carbon sources for soil microorganisms. These products can stimulate microbial growth and reproduction, thereby modifying the community structure and abundance of soil microorganisms (Braunack et al., 2015). Therefore, BP films tend to exert a more favorable influence on soil microbial populations compared with traditional PE films.

Based on the degradation characteristics of plastic films at different sites and soil microbial indicators, a 0.01 mm BP film was identified as the most suitable option for cotton fields, balancing low cracking rates during the seedling stage with soil environmental compatibility. A comprehensive evaluation of degradable films should also incorporate crop yield, soil moisture, temperature, and plant growth parameters (Dong et al., 2013; Zhao et al., 2023). Furthermore, to fully assess the environmental benefits and long-term impacts of BP films, additional research and continual monitoring are required, taking into account the interactions of multiple influencing factors. It should be also noted that different film types (e.g., biodegradable vs. conventional PE film) vary in cracking dynamics and degradation products, which may in turn exert distinct effects on soil microbial environment (Liu et al., 2010).

### 4.2 Differences in soil microorganisms in two sites

Geographical location, climate, soil type, and texture differed between the experimental sites, potentially influencing the soil microbial environment. Changji, located in the northeastern of Xinjiang, is characterized by a typical continental arid climate with cold winters, hot summers, and large diurnal temperature variations. Terrain effects cause notable climate variation from south to north, with higher summer precipitation in the south and pronounced desert conditions in the north (Chen C. B. et al., 2023). Hutubi County, situated in central northern Xinjiang, also experiences a temperate continental climate, but its varied altitudes result in marked climatic differences northern and southern areas. Although the soil types in both regions are complex, they are generally moderate and suitable for crop cultivation.

In this study, no substantial differences in soil microbial abundance were observed between the two sites under different mulching treatments. In both locations, the CK treatment supported the highest number of bacteria and actinomycetes, while the T2 treatment had the highest fungi abundance. Microbial diversity exhibited similar patterns across sites: the Shannon Index and Simpson Index showed no significant variation throughout the growth period, whereas the McIntosh Index displayed relatively pronounced changes (Figures 5, 6).

## 5 Conclusion

In both the Changji and Hutubi cotton fields, the overall trends in microbial population dynamics were generally consistent across treatments. Bacterial and actinomycete abundances were highest under traditional polyethylene film, whereas fungal abundance peaked under 0.012 mm biodegradable film. With increasing incubation time, the total metabolic activity of soil microorganisms toward carbon sources increased markedly after 24 h under all treatments. Across the entire cotton growth period, microbial diversity indices showed no significant changes, suggesting that mulching type had limited impact on soil microbial diversity. The cracking rate of the film and the local environmental conditions were key factors influencing the soil microbial community. Based on these results, a 0.01 mm biodegradable film appears to be the most suitable option for cotton cultivation, as it maintains a low cracking rate during the seedling stage while being more environmentally friendly than conventional plastic film. Nevertheless, the practical application of biodegradable films should be evaluated comprehensively, taking into account yield, soil moisture, temperature, and crop growth performance. To fully assess their environmental benefits, further research and long-term field monitoring are required.

## Data Availability

The raw data supporting the conclusions of this article will be made available by the authors, without undue reservation.
